# Announcement: *4th* International Symposium on Applied Bioinorganic Chemistry

**DOI:** 10.1155/MBD.1996.171

**Published:** 1996


					4th INTERNATIONAL
SYMPOSIUM ON APPLIED
BIOINORGANIC CHEMISTRY
including THE CARMAN NATIONAL PHYSICAL CHEMISTRY CONFERENCE.
Cape Town, South Africa
1-4 April 1997
LOCAL ORGANIZING COMMITTEE
H. Andrews, S. Bourne, A. Crouch, T. Demana, I. Green, G.E. Jackson, N. Jarvis, K.R. Koch,
P.W. Linder, I.M. Moodie, S. Wilson
INTERNATIONAL ADVISORY COMMITTEE
I. Bertini (Italy), A. Buller (USA), Anbang Dai (China), Y. Kidani (Japan), Liplin Koh (Sin-
gapore), Wang Kui (China), V.G. KumarDas (Malaysia), Jiaxi Lu (China), Jiazuan Ni (China),
ChungkWong Poon (Hong Kong), E. Ralilla (Philippines), J. Reedijk (Netherlands), P. Saltman
(USA), H. Sigel (Switzerland), Y. Sugiura (Japan), G. Sykes (UK), J. Webh (Australia), U.
Weser (Germany), P. Wilairat (Thailand), D.R. Williams (UK), K.B. Yatzimirskii (Ukraine)
This conference is to be held in the beauliful surroundings of Cape Town, South Africa, from
1 to 4 April 1997. The aim is to provide a forum for discussing the development and applica-
tions of bioinorganic chemistry with an emphasis on improving the quality of mankind's
existence.
Scientific Programme
Thc scientific programme comprising lectures and posters will include contributions from
prominent international researchers. Themes will include metal-containing drugs,
radiopharmaceuticals, toxicology, biologically active chelates and macrocycles, metallo-pro-
teins and enzymes, biominerals and new materials, biotechnology and environmental appli-
cations.
Laneuaee
In the interests of ensuring free exchange of information and ideas, the proceedings will he
conducted in English. Regrettably, no translation facilities will be available.
171
Venue
Greater Cape Town hosts a populalion of 2.5 million people and its spectacular harbours are
regarded as the gateway to Africa. The sites of the older harbours accommodate the newly
constructed Victoria and Albert Waterfront, a popular delight for both tourists and local peo-
ple. The Majestic Table Mountain forms a backdrop to down-town Cape Town, which is situ-
ated at the northern end of the scenic Cape Peninsula. The westcrn and eastern coasts of the
Peninsula abound with attractive beaches located on both the colder Atlantic and the warmer
Indian Ocean shores. Some 50 km distant lies the picturesque wine-producting town of
Stellenbosch nestling against the slopes of the Jonkershoek mountains.
Social Events
A variety of Social Events and tours will be organised for delegates and accompanying per-
sons. These events will be designed to maximise informal contact between participants as
well as to introduce them to the traditional Cape cuisine and wines.
Second circular
If you would like to receive the second circular, which will contain details regarding the
scientific and social programmes as well as accommodation, please complete and return the
reply form to
Fourth ISABC Conference Secretariat
Medical Research Council
P. O. Box 19070
Tygerberg, 7505, South Africa
THE FOURTH INTERNATIONAL SYMPOSIUM
ON APPLIED BIOINORGANIC CHEMISTRY
Cape Town 1st 4th April 1997
Replyform to 1st Circular Onlypersons who reply to this circular wll he put on the mailing
listfor the second circular.
Surname:
First Name(s): Title:
Address:
Postal code
Tel No.: Fax No.:
E-Mail:
I would like to present a Paper/Poster. (Delete which is not applicable).
My paper will be on thefollowing theme:
172

				

